# Co-circulation of two SARS-CoV-2 variant strains within imported pet hamsters in Hong Kong

**DOI:** 10.1080/22221751.2022.2040922

**Published:** 2022-02-27

**Authors:** Kin-Hang Kok, Shuk-Ching Wong, Wan-Mui Chan, Lei Wen, Allen Wing-Ho Chu, Jonathan Daniel Ip, Lam-Kwong Lee, Ivan Tak-Fai Wong, Hazel Wing-Hei Lo, Vincent Chi-Chung Cheng, Alex Yat-Man Ho, Bosco Hoi-Shiu Lam, Herman Tse, David Lung, Ken Ng Ho-Leung Ng, Albert Ka-Wing Au, Gilman Kit-Hang Siu, Kwok-Yung Yuen

**Affiliations:** a State Key Laboratory of Emerging Infectious Diseases, Carol Yu Centre for Infection, Department of Microbiology, Li Ka Shing Faculty of Medicine, The University of Hong Kong, Pokfulam, Hong Kong Special Administrative Region, People's Republic of China; b bCentre for Virology, Vaccinology and Therapeutics, Hong Kong Science and Technology Park, Hong Kong Special Administrative Region, People’s Republic of China;; c Infection Control Team, Queen Mary Hospital, Hong Kong West Cluster, Hong Kong Special Administrative Region, People’s Republic of China; d Department of Health Technology and Informatics, The Hong Kong Polytechnic University, Hong Kong Special Administrative Region, People’s Republic of China; e Department of Microbiology, Queen Mary Hospital, Hong Kong Special Administrative Region, People’s Republic of China; f Department of Pathology, Princess Margaret Hospital, Hong Kong Special Administrative Region, People’s Republic of China; g Department of Pathology, Hong Kong Children's Hospital, Hong Kong Special Administrative Region, People’s Republic of China; h Department of Pathology, Queen Elizabeth Hospital, Hong Kong Special Administrative Region, People’s Republic of China; i Centre for Health Protection, Department of Health, Hong Kong Special Administrative Region, People’s Republic of China

**Keywords:** Animal, coronavirus, hamster, interspecies, transmission, SARS-CoV-2

## Abstract

During the investigation of a pet shop outbreak of severe acute respiratory coronavirus 2 (SARS-CoV-2) with probable hamster-to-human transmission, the environmental and hamster samples in epidemiologically linked pet shops were found positive for SARS-CoV-2 Delta variant AY.127 strains which are phylogenetically closely related to patients and reported European strains. This interspecies’ spill-over has triggered transmission in 58 patients epidemiologically linked to three pet shops. Incidentally, three dwarf hamsters imported from the Netherlands and centralized in a warehouse distributing animals to pet shops were positive for SARS-CoV-2 spike variant phylogenetically related to European B.1.258 strains from March 2020. This B.1.258 strain almost disappeared in July 2021. While no hamster-to-human transmission of B.1.258-like strain was found in this outbreak, molecular docking showed that its spike receptor-binding domain (RBD) has a similar binding energy to human ACE2 compared to that of Delta variant AY.127. Therefore, the potential of this B.1.258-related spike variant for interspecies jumping cannot be ignored. The co-circulation of B.1.258-related spike variants with Delta AY.127, which originated in Europe and was not previously found in Hong Kong, suggested that hamsters in our wholesale warehouse and retail pet shops more likely have acquired these viruses in the Netherlands or stopovers during delivery by aviation than locally. The risk of human-to-hamster reverse zoonosis by multiple SARS-CoV-2 variants leading to further adaptive spike mutations with subsequent transmission back to humans cannot be underestimated as an outbreak source of COVID-19. Testing imported pet animals susceptible to SARS-CoV-2 is warranted to prevent future outbreaks.

## Introduction

Coronaviruses are positive-sense, single-stranded RNA viruses belonging to the family *Coronaviridae* in the order *Nidovirales* [1]. There are four genera in the family *Coronaviridae*: *Alphacoronavirus*, *Betacoronavirus*, *Gammacoronavirus*, and *Deltacoronavirus* [2]. Coronaviruses originating from mammals, especially bats and rodents, are generally considered the gene sources of *Alphacoronavirus* and *Betacoronavirus*, whereas avian coronaviruses the gene sources of most *Gammacoronavirus* and *Deltacoronavirus* [2]. The seven human-pathogenic coronaviruses known thus far, including the human coronaviruses HCoV-OC43, HCoV-229E, HCoV-NL63, and HCoV-HKU1, the highly virulent severe acute respiratory syndrome coronavirus (SARS-CoV-1) and Middle East respiratory syndrome coronavirus (MERS-CoV), the pandemic severe acute respiratory syndrome coronavirus 2 (SARS-CoV-2) have previously likely crossed species barriers to jump from mammalian animal species to humans [1,3,4].

While more than 70% of emerging human pathogens originate in animals, most involve wild animals and not pet animals [5]. Pet-related transmission of viruses to humans is relatively uncommon. Examples include lymphocytic choriomeningitis virus (LCMV) (from mice, hamtsers, and guinea pigs), rabies virus (from unvaccinated cats and dogs), B virus (from pet non-human primates), and cowpox virus (from pet rats) [6–9]. Sporadic cases of pet shop-related transmission of these zoonotic viruses have been reported occasionally, with the largest cluster having involved 181 symptomatic LCMV-infected patients in contact with hamsters sourced from a single distributor in the United States in 1974 [10]. However, pet shop-related outbreak of the pandemic SARS-CoV-2 has not been reported prior to our recent investigation [11]. During the investigation of this unusual cluster of probable hamster-to-human SARS-CoV-2 transmission due to the SARS-CoV-2 AY.127 variant, we incidentally found a hamster-related SARS-CoV-2 B.1.258-like spike variant. This study investigates this progressive outbreak and some genomic features of this newly identified hamster-associated SARS-CoV-2 spike variant.

## Materials and methods

### Epidemiological investigation and collection of human or hamster samples for virological testing

The patient data were collected in a standardised format by the Centre for Health Protection, Department of Health, the Hong Kong Special Administrative Region. The sex, age, vaccination status, residential address, contact history and the date of symptom onset or the date of reporting of asymptomatic cases were recorded for analysis. All preliminary positive clinical samples (deep throat saliva, nasal and throat swabs) for SARS-CoV-2 reported by private or hospital laboratories were confirmed by the Public Health Laboratory Services Branch (PHLSB) using in-house and/or commercial platforms of real-time RT–PCR assays (RdRp, E, N gene). The commercial platform used 600 ul of clinical samples for each cobas® SARS-CoV-2 test on cobas® 8800 and 300 ul of clinical samples for each Xpert® Xpress SARS-CoV-2 test on GeneXpert system according to the manufacturer’s instructions.

Samples confirmed positive would be screened for variant detection by different in-house single-nucleotide polymorphism (SNP) genotyping assay (N501Y, L452R and T478K) of the Spike (S) gene. L452R serves as a marker mutation for the variant Delta. L452R positive specimens were subject to whole-genome sequencing. Hamsters were euthanized by veterinarians of the Agriculture Fishery and Conservation Department (AFCD) before lung tissues, oral or anal swabs were collected for RT–PCR detection of SARS-CoV-2 and subsequent nanopore sequencing.

### Whole-genome sequencing

Direct whole viral genome sequencing of patients’ specimens was done using PCR tiling of SARS-CoV-2 virus with rapid barcoding protocol (Version: mrt_9127_v110_revH_14Jul2021) on Nanopore GridION MK1 (Oxford Nanopore Technologies), as we described previously [12]. Illumina sequencing was used for environmental and animal samples from pet shops. Bioinformatic analysis was performed with the recommended ARTIC bioinformatics workflow with minor modifications, as previously described [12,13].

### Viral sequences for analysis

The representative genome sequences of SARS-CoV-2 in this study were available at GISAID database (A: EPI_ISL_402125; B.1.1.7: EPI_ISL_601443; B.1.351: EPI_ISL_9002699; P.1: EPI_ISL_872192; B.1.617.2: EPI_ISL_3844030; B.1.1.529: EPI_ISL_8724747; B.1.258: EPI_ISL_1492308). Details of sequences are listed in Supplementary Table 1. The sequences of case-1, case-2, case-3 and one environmental sample have been described elsewhere (11). In this study, the full-length genome of case-6 (OM475730), case-7 (OM475731), case-8 (OM475729), case-9 (OM475732), case-12 (OM475733), case-13 (OM475736), case-14 (OM475734), case-17 (OM475735), case-25 (OM475739), case-30 (OM475740), case-31 (OM475737), case-33 (OM475738), cage-B-H20 (OM475741), cage-C-H10 (OM475742), cage-C-H11 (OM475743), cage-C-H12 (OM475744), NTS-S-2022-01-10 oral sample (OM475745), and NTS-S-2022-01-10 fecal sample (OM475746) have been submitted to GenBank database. The three dwarf hamster samples found in the warehouse were tested positive by RT–PCR, but no other whole-genome analysis could be done in the previous study due to low viral load (11). In this report, the partial spike nucleotide sequences of warehouse hamster L59 (CoV-19/Hong Kong/HKU-220204-L59/2022; EPI_ISL_9502991), L60 (hCoV-19/Hong Kong/HKU-220204-L60/2022; EPI_ISL_9502992) and L64 (hCoV-19/Hong Kong/HKU-220204-L64/2022; EPI_ISL_9502993) have been deposited into GISAID database.

### Genome characterization and phylogenetic analysis

A maximum-likelihood whole-genome phylogenetic tree was constructed using IQ-TREE2 [13], with the generalized time-reversible substitution model GTR + F+I as the best-predicted model by BIC. The option -czb was used to mask any unrelated substructures of the tree, with branch length representing a mutation count of < 1. The ultrafast bootstrap option was used with 1,000 replicates.

Phylogenetic tree of spike proteins inferred from 1000 replicates, was generated by the Neighbor-Joining method [14]. The number next to the branches indicated the percentage of replicate trees. The evolutionary distances were analysed by the JTT-G method in MEGA-X.

### Molecular docking and ACE2 binding affinity analyses

The ACE2 protein sequences of human (*Homo sapiens*; Q9BYF1), dwarf hamster (*Phodopus roborovskii*; A0A7T0PYW5), golden Syrian hamster (*Mesocricetus auratus*; A0A1U7QTA1), and European mink (*Mustela lutreola*; A0A7G6KLV1) were downloaded from the Uniprot database [15]. Multiple sequence alignment was constructed with MUSCLE [16]. The crystal structure of SARS-CoV-2 spike RBD and human ACE2 complex (code: 6M0J) was retrieved from the Protein Data Bank [17]. ACE2 structures of other animal species were generated by mutating the interface residues according to the multiple sequence alignment. The complex of ACE2 and RBD was built by superimposition with a 6M0J structure. All the ACE2-RBD complexes were locally refined with Rosetta relax and docking protocols. Interface-binding energies were estimated with the InterfaceAnalyzer application [18].

## Results

### Epidemiological investigation on a large outbreak chain due to the probable hamster-to-human transmission of SARS-CoV-2

**Figure 1.**
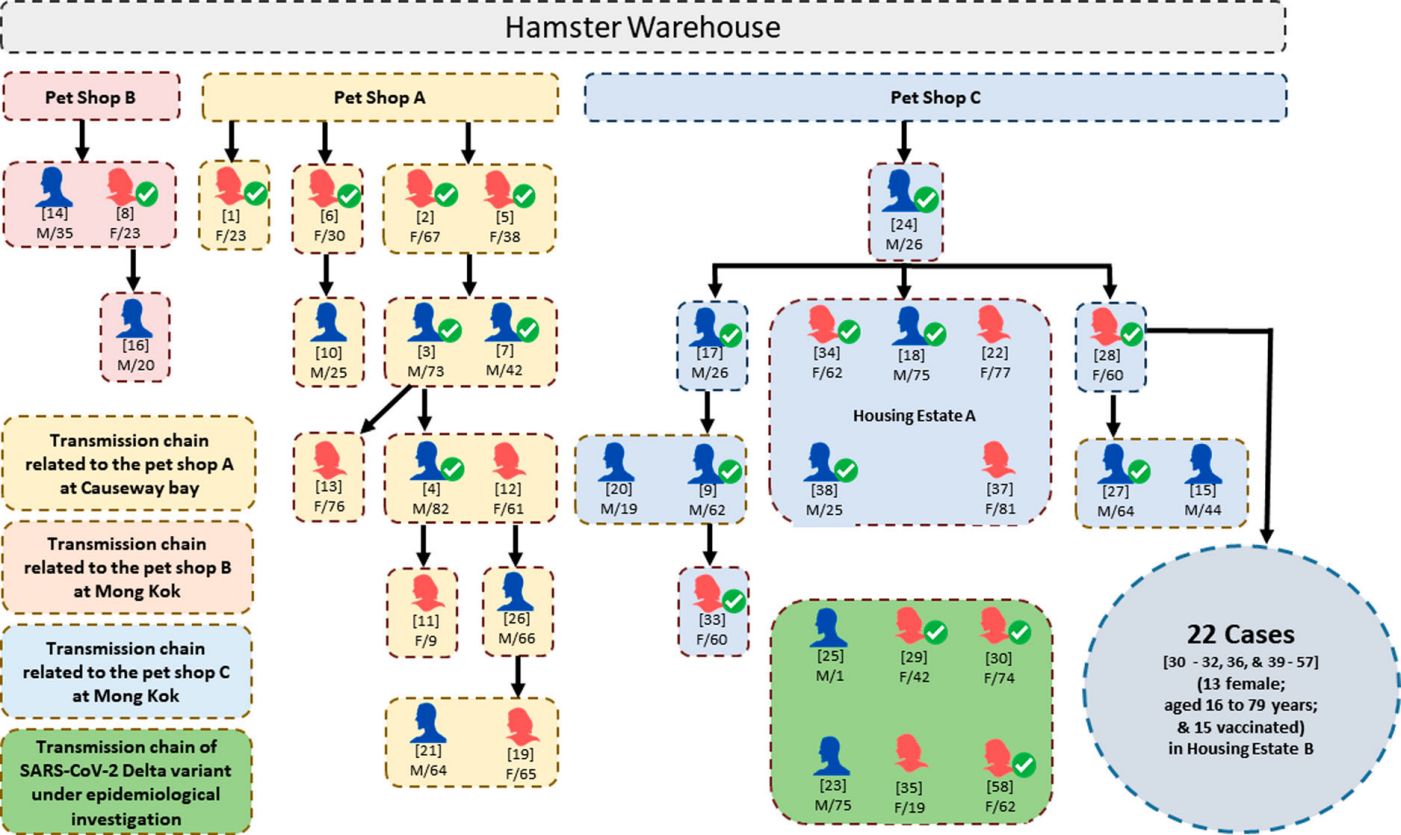
Transmission chain of a probable pet shop-related outbreak due to SARS-CoV-2 Delta AY.127 variant. Male and female laboratory-confirmed COVID-19 patients linked with this outbreak are represented by blue and red icons, respectively. The patients who had received at least two doses of inactivated or mRNA COVID-19 vaccines are indicated by ticks. The case numbers are represented by the numbers in square brackets above the sex and age of each patient. The transmission chain related to pet shop A involved transmission within family members and dining premises. The transmission chain related to pet shop B was limited. The transmission chain related to pet shop C involved transmission within family members and other residents in the same housing estate A and housing estate B.

**Figure 2.**
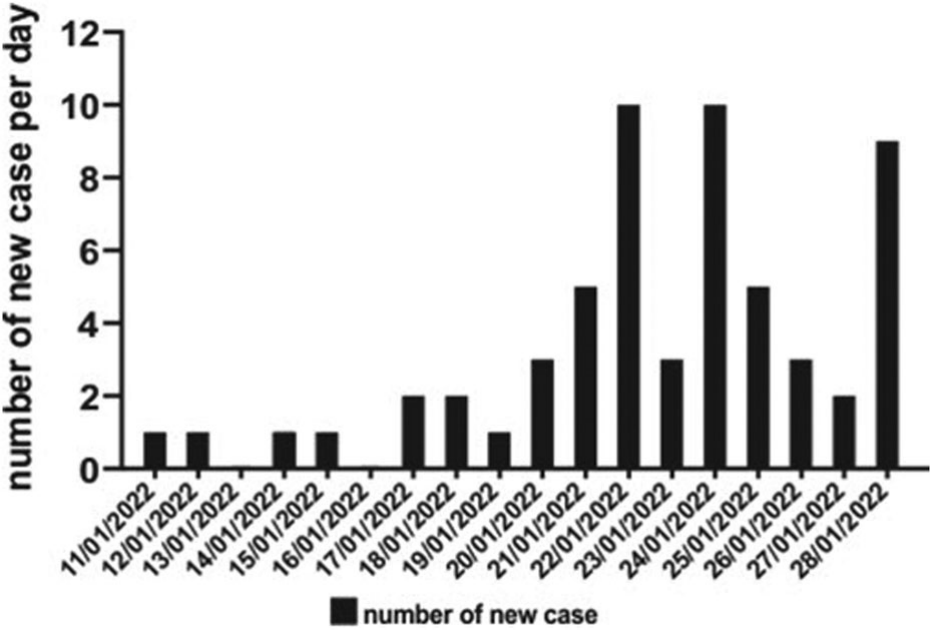
Epidemic curve of a probable pet shop-related outbreak of SARS-CoV-2 Delta AY.127 variant. A total of 58 COVID-19 cases were reported. The timeline represents the date of symptom onset for symptomatic cases or the date of reporting for asymptomatic COVID-19 cases.

Since our recent report of the first three human cases due to SARS-CoV-2 Delta AY.127 variant linked to this pet shop-related outbreak of COVID-19 [11], additional patients, who were epidemiologically linked to three retail pet shops, supplied by a single wholesale warehouse holding over a thousand hamsters were identified. A summary of the probable transmission chains and the symptom onset (symptomatic cases) or test reporting dates (asymptomatic cases) are depicted in Figure 1 and Figure 2, respectively. At the time of writing, 58 laboratory-confirmed human cases of SARS-CoV-2 AY.127 variant primarily linked to this outbreak were detected. There were 27 males and 31 females. The mean age was 48 years (range: 1–82). Thirty four of them (58.6%) had received at least two doses of inactivated (Sinovac or Sinopharm) or mRNA (Pfizer-BioNTech) COVID-19 vaccine. The first chain of transmission started with case 1, a 23-year-old shopkeeper at pet shop A. She had contact with her customer case 2 [11]. Three family members (cases 3, 5, and 7) of case 2 were subsequently infected and spread the infection to 7 other patients (cases 4, 11, 12, 13, 19, 21, and 26). The second chain of transmission originated from pet shop B leading to 3 cases (cases 8, 14, and 16). The third chain of transmission started with case 24, who acquired the infection from pet shop C and transmitted it to his 3 co-workers and 1 family member (cases 9, 17, 20, 33). Subsequently, he spread the infection to housing estate A (cases 18, 22, 34, 37, 38). Then his mother (case 28) got infected and transmitted the virus to 22 other cases in another housing estate B (cases30, 31, 32, 36, 39–57). There were 6 cases (cases 23, 25, 29, 30, 35, 58) of Delta AY.127 that were still under epidemiological investigation.

### Genomic characterization of hamster-associated SARS-CoV-2 outbreak

**Figure 3.**
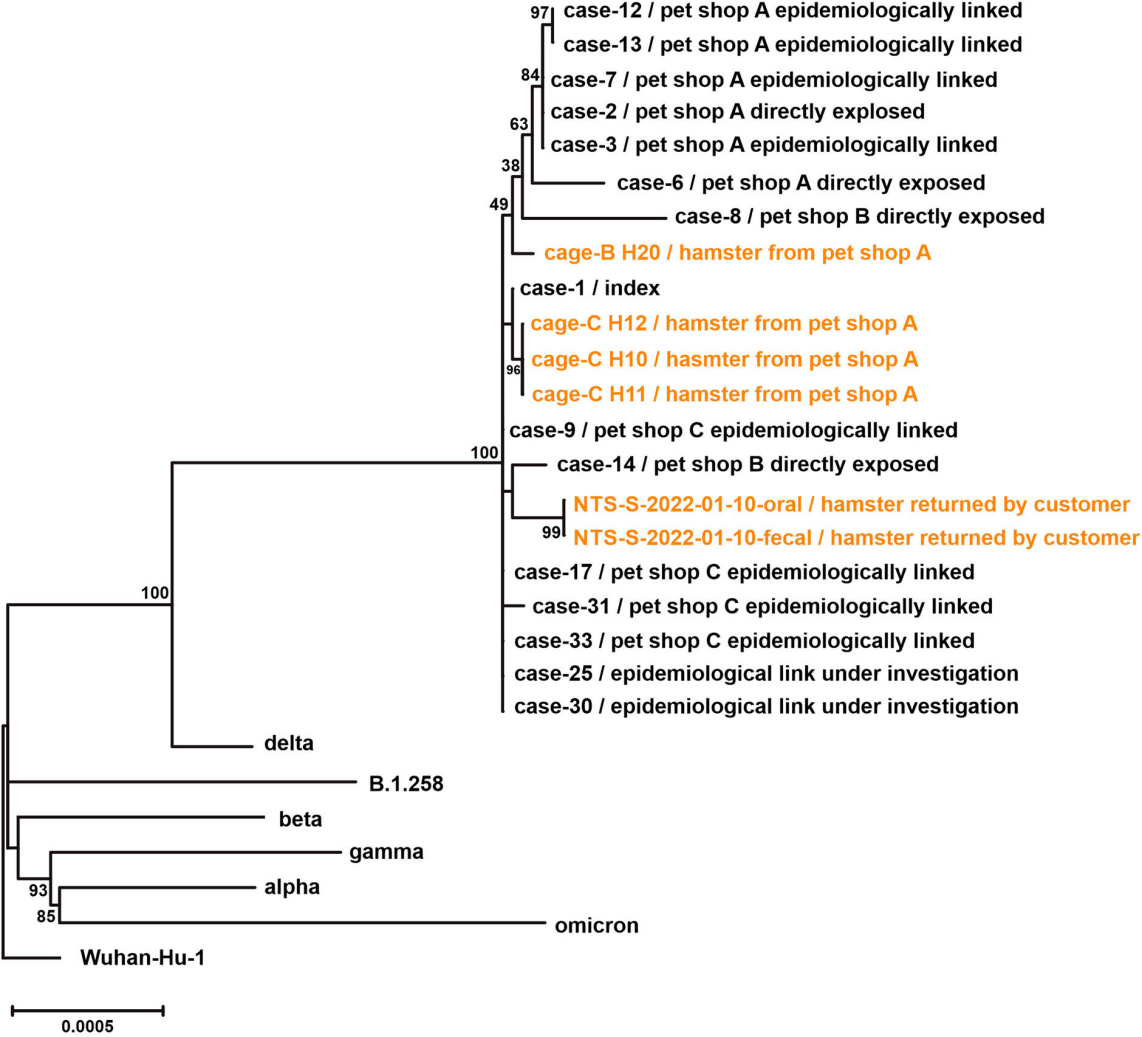
Phylogenetic tree of whole-genome sequences of SARS-CoV-2 found in patients, hamsters or environment of pet shops. Complete viral genome nucleotide sequences were aligned, and the phylogenetic tree was generated by Maximum Likelihood method and generalized time-reversible substitution model GTR + F+I. The number next to the branches indicated the bootstrap values representing the percentage of 1000 replicates. Full-length genomic sequences derived from 15 patient samples and six hamster swab samples, one representative genome sequence of original SARS-CoV-2 (Wuhan_Hu_1), five Variant of Concern strains (Alpha, B.1.1.7; Beta, B.1.351; Gamma, P.1; Delta, B.1.617.2; Omicron, B.1.1.529) and SARS-CoV-2 lineage B.1.258 were included in the analysis.

The whole viral genome sequences were obtained from 15 patients, directly exposed to or epidemiologically linked to pet shops A, B and C. All these cases were of Delta AY.127 and were phylogenetically related to six hamster swab samples, which included four samples collected from hamsters in the pet shops and two samples of a hamster returned by a pet shop customer. (Figure 3).

Among the four hamster samples from pet shop A, three harboured a novel non-synonymous mutation, H49Y, in spike. Interestingly, this mutation was also identified in the index case (case 1), a saleslady in the same pet shop, suggesting that this mutation may be associated with the initial interspecies spill-over (Supplementary Table 2). In addition, another non-synonymous mutation in NSP3 (S1428L) was commonly found in a hamster returned by a customer and in two cases directly exposed to pet shop B (case 8 and case 14). We believed that these two cases might have acquired the virus from hamsters that belonged to the same batch of the returned one. Since this mutation has not been found in the major Variants of Concern (Alpha, Beta, Gamma, Delta, and Omicron), they may be new adaptative substitutions found amongst the pet shop hamsters (Supplementary Table 2).

**Figure 4.**
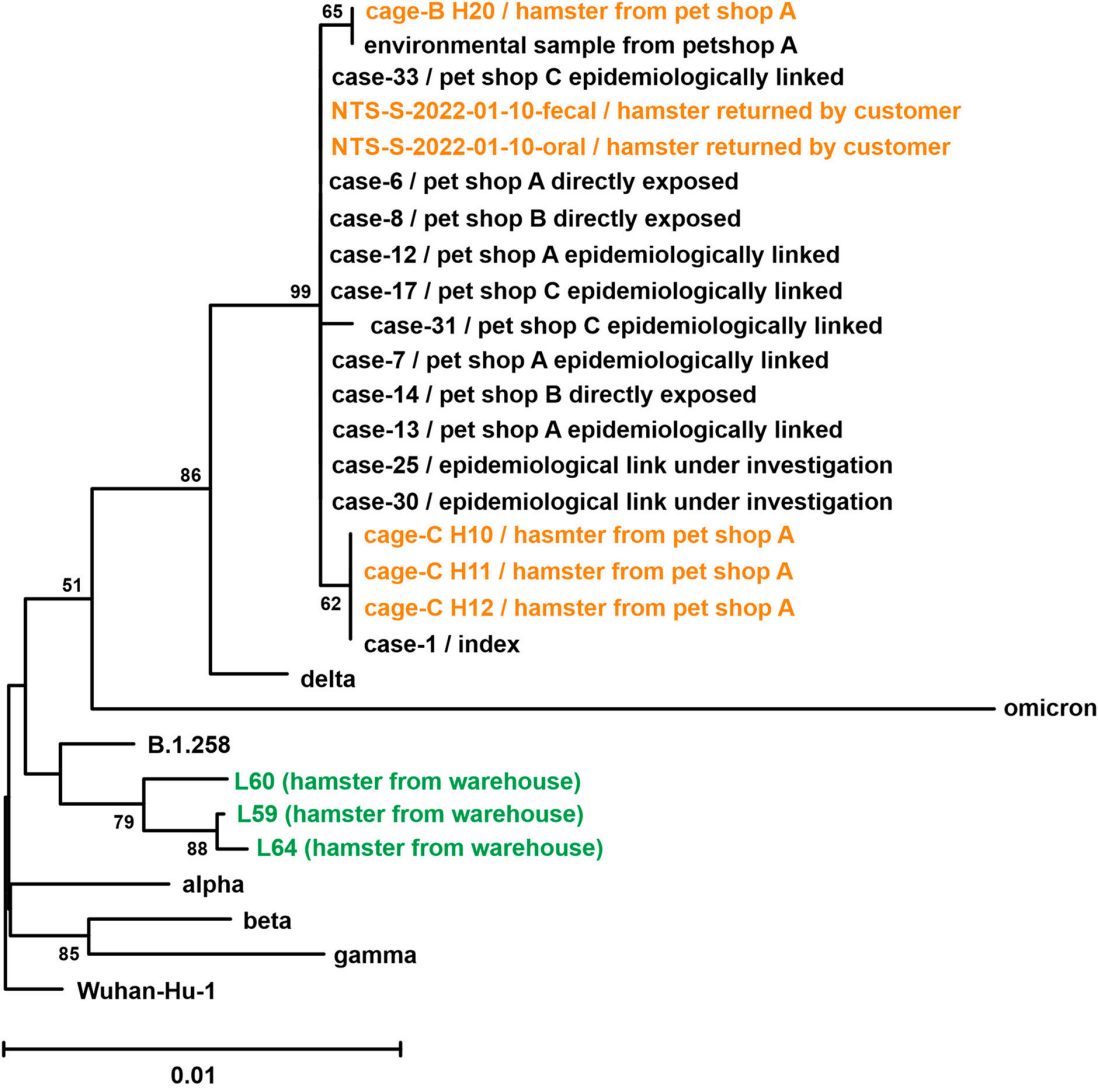
Phylogenetic tree of SARS-CoV-2 spike amino acid sequences from patients, the environment of the pet shop, hamster of a pet shop or wholesale warehouse. Amino acid sequences of spike protein were aligned, and the bootstrap consensus tree was generated by the Neighbor-Joining method. Spike sequences derived from lung tissue of three hamsters (L59, L60 and L64), representative spike amino acid sequence of original SARS-CoV-2 (Wuhan_Hu_1), Variant of Concern strains (Alpha, B.1.1.7; Beta, B.1.351; gamma, P.1; Delta, B.1.617.2; Omicron, B.1.1.529), one closest strain to hamster spike sequences (B.1.258) and spike sequences derived from 12 patient samples and six hamster swab samples were included in the analysis. Detailed information of reference sequences (accession ID, location and virus name) was listed in Supplementary Table 1.

The phylogenetic analysis of spike amino acid sequences revealed that all samples obtained from pet shops also clustered with the Delta AY.127 variant, but the three lung tissue samples collected from dwarf hamsters located in the wholesale warehouse were all similar to B.1.258, which is the variant circulating in Europe from March 2020 to December 2021, prior to the emergence of the Alpha variant (B.1.1.7) [19] (Figure 4 and Supplementary Table 3). The first B.1.258 was reported in March 2020 from England. Subsequent cases were reported throughout Europe, including the Netherlands [20,21]. At the time of writing, 5,596 B.1.258 cases were reported on GISAID, with the highest percentage of prevalence reported from Cyprus. Notably, 315 cases were reported from the Netherlands. The reports of B.1.258 cases have markedly decreased in GISAID after July 2021.

### Molecular docking analysis

**Figure 5.**
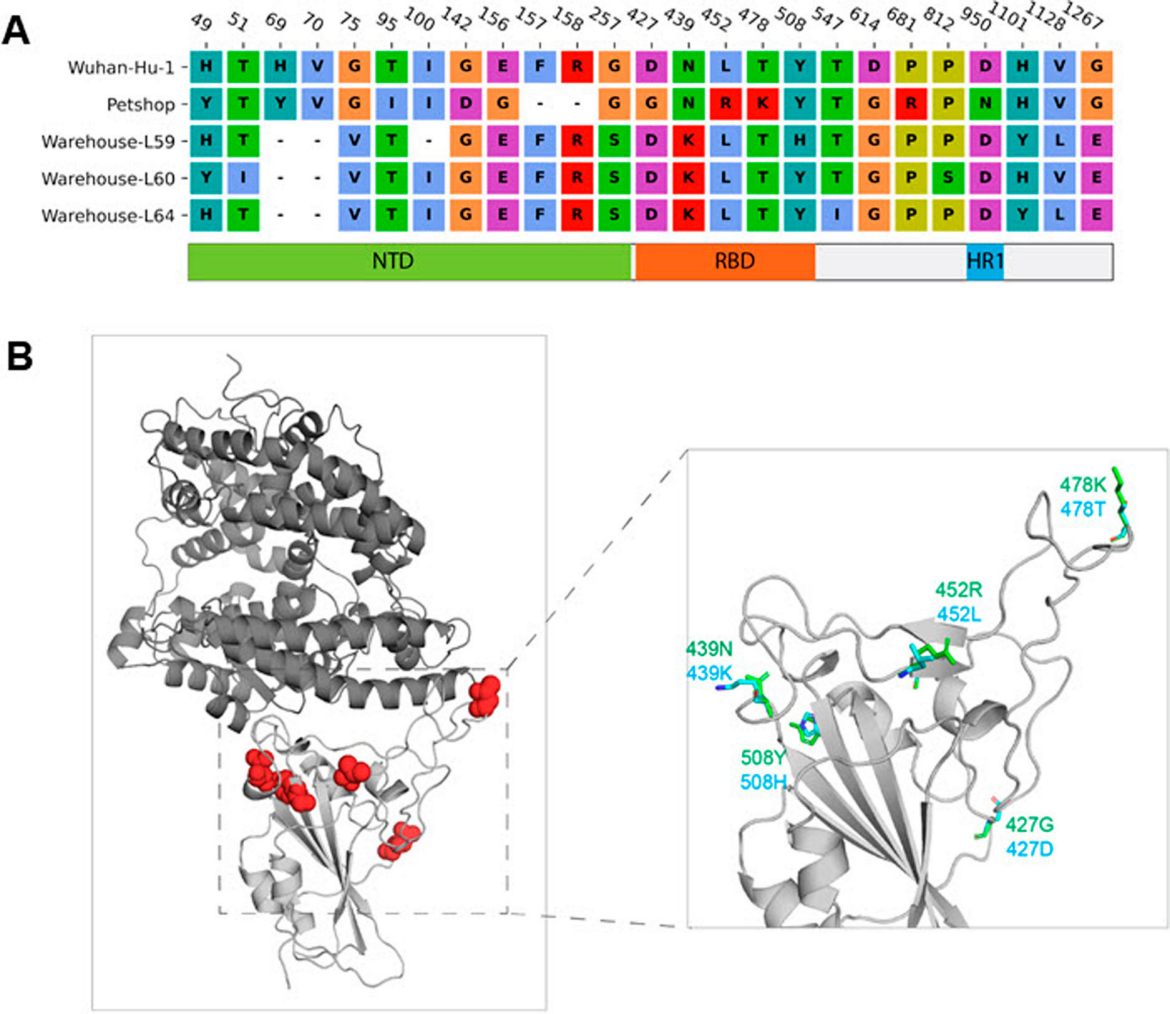
Mutations of pet shop strains and warehouse strains L59, L60 and L64 compared to Wuhan-Hu-1. (A) Sequence alignment of spike protein mutation sites. The corresponding domains of the mutations were indicated. (B) Locations of the RBD mutations in three-dimensional RBD-ACE2 complex structure. ACE2 and RBD were shown in the dark and light grey cartoon, respectively. The mutation sites were highlighted with a red sphere. The predicted side-chain conformations of RBD with mutations were shown in green and cyan stick representation.

**Figure 6.**
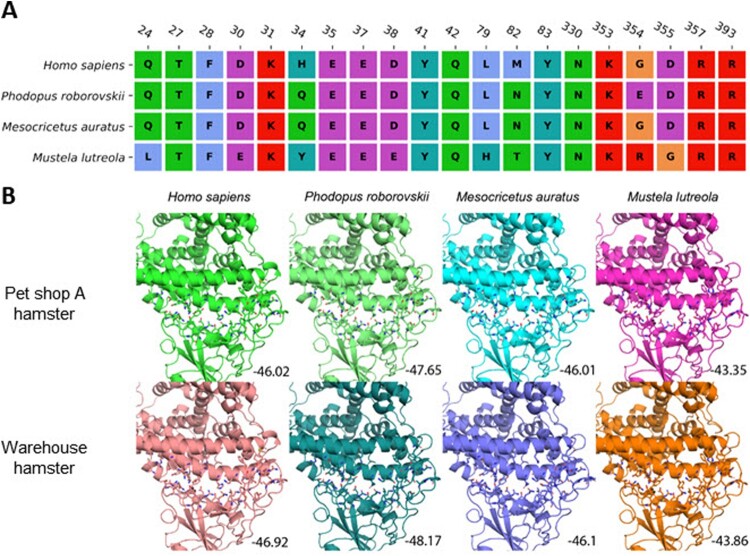
Protein-protein docking between RBD and ACE2. (A) Sequence alignment of ACE2 residues in human (*Homo sapiens*), dwarf hamster (*Phodopus roborovskii*), golden Syrian hamster (*Mesocricetus auratus*) and European mink (*Mustela lutreola*) interacting with SARS-CoV-2 spike protein RBD. (B) Interactions between ACE2 and SARS-CoV-2 spike RBD from the pet shop and warehouse hamster virus. Interacting amino acids are shown in stick representation. Predicted binding scores are indicated in the bottom corner.

According to the protein sequence alignment, mutations were found at 27 sites of the spike protein in the pet shop A hamster strain and the warehouse hamster strains compared with the wild-type Wuhan-Hu-1 strain. While most mutations (14/27) occurred in the NTD of the spike, 5 mutations were found in the RBD (Figure 5(A)). However, none of these 5 mutations outside the receptor-binding interface are directly involved in the receptor binding based on the three-dimensional structure analysis of the ACE2-RBD complex (Figure 5(B)). Since the mutations reside on the surface of spike RBD, we speculated that the conformation of RBD will not be altered significantly. To confirm this speculation, the RBD structures of the pet shop A strain and the warehouse L59 hamster strain were predicted on the basis of wild-type structure. Protein–protein docking was conducted to estimate the binding affinity between the RBDs of different strains and ACE2 proteins of humans (*Homo sapiens)*, dwarf hamster (*Phodopus roborovskii*), Golden Syrian hamster (*Mesocricetus auratus)*, and European mink (*Mustela lutreola)*. As shown in Figure 6, the pet shop A hamster strain and warehouse L59 hamster strain RBDs bind to human and both hamster ACE2 proteins with comparable affinities (between −46 and −48), due to the high similarity of ACE2 interface residues. However, the binding to mink ACE2 was relatively weaker (around −43) due to less compatible interface residues.

## Discussion

We have recently found that staff and customers of a pet shop were infected by a Delta SARS-CoV-2 AY.127 variant which is also phylogenetically almost identical to the genomes of isolates from hamsters and environmental samples in this pet shop [11]. Notably, this AY.127 was circulating in Europe but not in Hong Kong. Furthermore, the hamsters of the wholesale warehouse hamsters were infected by another SARS-CoV-2 spike variant most closely related to B.1.258, which does not belong to the known Variants of Concern. This relatively uncommon variant was first reported in England and circulated in Europe prior to the emergence of the Alpha variant (B.1.1.7) [20,21]. This B.1.258 has almost disappeared according to the SARS-CoV-2 genome surveillance of GISAID in July 2021. Moreover, this B.1.258-like spike variant strain contains several non-synonymous mutations in the spike, including the H69/V70 deletion and D614G substitution. While we have recently documented that the pet-shop Delta AY.127 variant can be transmitted from hamster to humans during the early part of his outbreak [11], the ability of this B.1.258-related spike variant strain to jump to humans remains uncertain as no human B.1.258 cases were found in this outbreak. As every confirmed case in Hong Kong is hospitalized, and the genome of their isolate is sequenced as long as the viral load is high enough, it is unlikely that the hamster associated Delta AY.127 or the B1.258-like virus was circulating in the Hong Kong population before this outbreak Therefore, an undetected local transmission chain in our population leading to the infection of the hamsters in the pet shop is also unlikely to be the source of this outbreak. Molecular docking analysis showed that this B.1.258-related spike variant has a similar degree of binding affinity to human ACE2 as that of the pet shop hamster Delta AY.127 strain but has much less affinity for mink ACE2 as control. This may indicate its potential for interspecies transmission from hamster to humans, as in the case of the pet shop Delta AY.127 strain. This finding is not unexpected as none of the amino acid substitutions in this B.1.258-related variant are positioned at the binding interface between the human ACE2 and spike RBD. Our findings suggested that transcontinental spread of non-local SARS-CoV-2 strains belonging to more than 1 variant is possible through imported pet animals.

SARS-CoV-2 has a wide species tropism as demonstrated in natural and laboratory settings [22]. Pet animals such as dogs, cats, rabbits and ferrets; zoo animals such as feline species, hippos, hyenas and gorillas; farm animals such as mink; and wild animals such as bank voles, racoon dogs, tree shrews, white-tail deer, Egyptian fruit bats, Asian small-clawed otters can all be infected by SARS-CoV-2 [23–26]. We have previously established the hamster model as the first physiological small animal model of COVID-19 [27]. Hamster-to-hamster transmission has been documented by direct contact, indirect contact through fomites, or non-contact routes by exhaled aerosol [27–29]. Furthermore, we have previously demonstrated that non-rodent-adapted SARS-CoV-2, such as alpha and beta variants with N501Y, can infect *Mus musculus* and/or *Rattus norvegicus* [30]. But despite the high susceptibility of hamsters to SARS-CoV-2, no transmission from hamsters to humans or other animals has been previously documented.

The on-going pet shop-related outbreak in Hong Kong since early January 2022 has already affected at least 58 patients. A hamster culling operation was launched at pet shops and the wholesale warehouse to stop the multiple chains of transmission. While there is no conclusive evidence on the origin of this Delta AY.127, the close genomic relationship of the pet shop-related strain to the European strains, including those from the Netherlands, suggested that the imported hamsters might have acquired these virus strains from the Netherlands or the stopover during delivery by aviation, and less likely from an infected incoming traveller who started a cryptic transmission chain from the wholesale warehouse to the retail pet shops, amongst which three of these pet shops had epidemiologically linked human cases. Although many instances of reverse zoonosis from human to pet, zoo, or farm animals were reported, only further transmission from mink back to human has been reported in many European farms where tens of thousands of mink were reared closely together. SARS-CoV-2 infection of minks was reported in the Netherlands in early 2020 [31]. Mink-associated outbreaks of animal-to-human transmission of SARS-CoV-2 were also reported in other parts of Europe [25]. The acquisition of COVID-19 in the pet shop setting with a much lower number of hamsters might suggest that other biological or environmental factors may facilitate better SARS-CoV-2 transmission from hamster to human compared with the mink farm setting.

The exact incubation period and doubling interval of SARS-CoV-2 in hamsters at the pet shop or warehouse setting areuncertain. Infected hamsters may be asymptomatic upon low virus dose exposure, and most of them would recover even with high dose exposure and clinical disease [27,32,33]. The continuous chain of transmission, especially with topping up by new batches of freshly imported and susceptible hamsters, can sustain the chain of transmission, which can last very long until all the hamsters are infected and recovered. The process may continue for months to infect the thousands of imported hamsters. Thus, the finding of quite several substitutions between strains of these two variants, AY.127 and B.1.258-like, circulating within these imported hamsters is not unexpected because adaptive mutations may occur rapidly upon interspecies jumping from animal-to-human or human-to-animal, respectively. The results of these adaptive mutations may change the virulence, antigenicity, and transmissibility of these hamster SARS-CoV-2 unpredictably. Hence, there was an urgent need to stop this chain of transmission by the Hong Kong Government that decided to cull all pets in the pet shop and warehouse due to limited alternative measures. The other option is to isolate all hamsters in a biosafety level 3 laboratory so that no virus can leak out into the community. The animals can be repeatedly tested till negative for at least 21 days before release. However, no such facility and manpower were available. Our local experience showed that just one imported case leaking into our community (36) could spark a major wave of infection affecting thousands of people. Therefore, we must ensure that this imported hamster associated with Delta AY.127 cannot gain a foothold in Hong Kong. Although another option is to enforce on-site quarantine and testing at pet shops and warehouses, this option would only be safe if most of our local population is fully vaccinated because inactivated and mRNA vaccines used in our locality are quite effective for the Delta variant. However, the overall vaccination rate in our whole population was still less than 70% and less than 30% for the high-risk elderly home residents at that juncture. Their low vaccination rate rendered them especially vulnerable to this Delta variant in case leaking occurs with on-site quarantine and testing. If most of our population is already vaccinated, the resulting morbidity and mortality associated with such a leak would be unlikely to overwhelm our healthcare service. Thus on-site quarantine and testing of hamsters cannot be recommended to control this outbreak. To prevent further such pet animal-related outbreaks, testing of imported pet animals susceptible to SARS-CoV-2 by RT–PCR and serology would be warranted.

Our study was limited by the availability of samples from the warehouse and the affected retail pet shop A. To get a complete picture of the genetic diversity of hamster-associated SARS-CoV-2 would require more animal samples from all 35 pet shops in Hong Kong. Due to the low viral load found in the lung tissues of the warehouse hamsters, complete genome sequences are not available for molecular dating to ascertain when this hamster-associated B.1.258-like spike variant arises. More viral genomic surveillance for hamster or pet animal-associated SARS-CoV-2 is warranted.
